# *N*-(2-Chloro­phen­yl)-2-({5-[4-(methyl­sulfan­yl)benz­yl]-4-phenyl-4*H*-1,2,4-triazol-3-yl}sulfan­yl)acetamide

**DOI:** 10.1107/S1600536811027565

**Published:** 2011-07-16

**Authors:** Hoong-Kun Fun, Chin Sing Yeap, K Manjunath, D. Jagadeesh Prasad, Boja Poojary

**Affiliations:** a X-ray Crystallography Unit, School of Physics, Universiti Sains Malaysia, 11800 USM, Penang, Malaysia; b Department of Chemistry, Mangalore University, Karnataka, India

## Abstract

In the title mol­ecule, C_24_H_21_ClN_4_OS_2_, the central 1,2,4-triazole ring forms dihedral angles of 89.05 (9), 86.66 (9) and 82.70 (10)° with the chloro-substituted benzene ring, the methyl­sulfanyl-substituted benzene ring and the phenyl ring, respectively. In the crystal, mol­ecules are linked into sheets parallel to (100) by inter­molecular N—H⋯N and weak C—H⋯O hydrogen bonds.

## Related literature

For general background to and applications of 1,2,4-triazole derivatives, see: Holla *et al.* (2002 ▶, 2003 ▶); Rudnicka *et al.* (1986 ▶); Burch & Smith (1966 ▶); Kalyoncuoglu *et al.* (1992 ▶); Mhasalkar *et al.* (1970 ▶); Mir *et al.* (1970 ▶).
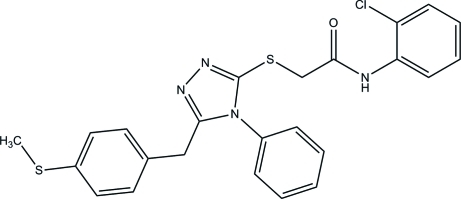


## Experimental

### 

#### Crystal data

C_24_H_21_ClN_4_OS_2_
*M* *_r_* = 481.02
Monoclinic, 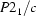

*a* = 14.2542 (7) Å
*b* = 16.3273 (9) Å
*c* = 10.1584 (6) Å
β = 96.372 (1)°
*V* = 2349.6 (2) Å^3^
*Z* = 4
Mo *K*α radiation
μ = 0.37 mm^−1^
*T* = 297 K
0.52 × 0.27 × 0.22 mm

#### Data collection

Bruker APEXII DUO CCD diffractometer
Absorption correction: multi-scan (*SADABS*; Bruker, 2009 ▶) *T*_min_ = 0.832, *T*_max_ = 0.924
29877 measured reflections
7900 independent reflections
5235 reflections with *I* > 2σ(*I*)
*R*_int_ = 0.028

#### Refinement

*R*[*F* ^2^ > 2σ(*F* ^2^)] = 0.049
*wR*(*F* ^2^) = 0.159
*S* = 1.05
7900 reflections
290 parameters
H-atom parameters constrained
Δρ_max_ = 0.53 e Å^−3^
Δρ_min_ = −0.45 e Å^−3^

### 

Data collection: *APEX2* (Bruker, 2009 ▶); cell refinement: *SAINT* (Bruker, 2009 ▶); data reduction: *SAINT*; program(s) used to solve structure: *SHELXTL* (Sheldrick, 2008 ▶); program(s) used to refine structure: *SHELXTL*; molecular graphics: *SHELXTL*; software used to prepare material for publication: *SHELXTL* and *PLATON* (Spek, 2009 ▶).

## Supplementary Material

Crystal structure: contains datablock(s) global, I. DOI: 10.1107/S1600536811027565/lh5283sup1.cif

Structure factors: contains datablock(s) I. DOI: 10.1107/S1600536811027565/lh5283Isup2.hkl

Supplementary material file. DOI: 10.1107/S1600536811027565/lh5283Isup3.cml

Additional supplementary materials:  crystallographic information; 3D view; checkCIF report

## Figures and Tables

**Table 1:** Hydrogen-bond geometry (Å, °)

*D*—H⋯*A*	*D*—H	H⋯*A*	*D*⋯*A*	*D*—H⋯*A*
N1—H1*N*1⋯N3^i^	0.94	2.04	2.9787 (19)	174
C8—H8*A*⋯O1^ii^	0.97	2.52	3.147 (2)	123
C11—H11*B*⋯O1^iii^	0.97	2.47	3.416 (2)	165

Symmetry codes: (i) 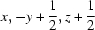; (ii) 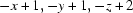; (iii) 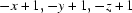.

**Fig. 1.**
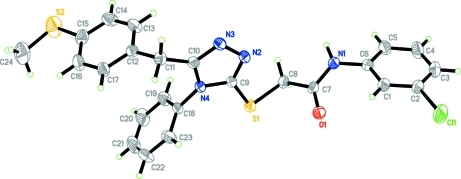
The molecular structure of (I), with atom labels and 30% probability ellipsoids for non-H atoms.

**Fig. 2.**
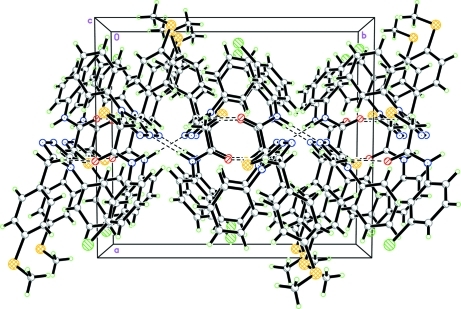
The crystal packing of (I), showing the molecules linked into a double sheets parallel to (1 0 0). Hydrogen bonds are shown as dashed lines.

## supplementary crystallographic information

### Comment

1,2,4-Triazole compounds show a broad spectrum of biological activities, possibly due to the presence of the N–C–S group (Holla *et al.*, 2002). Among the 1,2,4-triazoles, the mercapto-thione-substituted 1,2,4-triazole ring systems have been well studied and so far, a variety of biological activities have been reported for a large number of their derivatives, such as antibacterial (Burch & Smith, 1966), antifungal (Kalyoncuoglu *et al.*, 1992), antitubercular (Mir *et al.*, 1970), antimycobacterial (Rudnicka *et al.*, 1986), anticancer (Holla *et al.*, 2003) and hypoglycemic properties (Mhasalkar *et al.*, 1970). In view of the above findings, and in continuation of our earlier work on the synthesis and biological activity of triazoles and their derivatives, we have synthesized a series of 1,2,4-triazole derivatives *via* joining a 1,2,4-triazole and acylamide group together in the same molecule and have studied their biological activities. As part of this research we have determined the crystal structure of th title compound.

The molecular structure of the title compound (I) is shown in Fig. 1. The central 1,2,4-triazole ring forms dihedral angles of 89.05 (9), 86.66 (9) and 82.70 (10)° with the three benzene rings (C1–C6, C18–C23 and C12–C17). In the crystal, molecules are linked into double sheets parallel to (1 0 0)  by intermolecular N1—H1N1···N3^i^, C8—H8A···O1^ii^ and C11—H11B···O1^iii^ hydrogen bonds (Table 1, Fig. 2).

### Experimental

A equimolar mixture of 5-[4-(methylthiobenzyl]-4-phenyl-4*H*-[1,2,4]-triazole-3-thione (0.01 mol), 2-Chloro-*N*-(2-chlorophenyl)acetamide (0.01 mol) and dry potassium carbonate (0.01 mol) were refluxed for 6 h in 20 ml of absolute alcohol and excess of solvent was removed by distillation under reduced pressure. After cooling to room temperature, the reaction mixture was poured into 50 ml of water. The product which precipitated was filtered off, washed with methanol and dried. The crude product was re-crystallized from ethanol.

### Refinement

The N-bound hydrogen atom was located from difference Fourier map and included in a riding-model approximation with N-H = 0.94Å and *U*_iso_(H) = 1.2*U*_eq_(N). All C-bound hydrogen atoms were positioned geomatrically [C–H = 0.93–0.97 Å] and refined using a riding model, with *U*_iso_(H) = 1.2 or 1.5 *U*_eq_(C). A rotating-group model were applied for methyl groups. Two reflections, (-1 1 1), and (6 6 4), were omitted.

### Crystal data

**Table:**

C_24_H_21_ClN_4_OS_2_	*F*(000) = 1000
*M**_r_* = 481.02	*D*_x_ = 1.360 Mg m^−^^3^
Monoclinic, *P*2_1_/*c*	Mo *K*α radiation, λ = 0.71073 Å
Hall symbol: -P 2ybc	Cell parameters from 7952 reflections
*a* = 14.2542 (7) Å	θ = 2.5–30.4°
*b* = 16.3273 (9) Å	µ = 0.37 mm^−^^1^
*c* = 10.1584 (6) Å	*T* = 297 K
β = 96.372 (1)°	Block, brown
*V* = 2349.6 (2) Å^3^	0.52 × 0.27 × 0.22 mm
*Z* = 4	

### Data collection

**Table:**

Bruker APEXII DUO CCD diffractometer	7900 independent reflections
Radiation source: fine-focus sealed tube	5235 reflections with *I* > 2σ(*I*)
graphite	*R*_int_ = 0.028
φ and ω scans	θ_max_ = 31.7°, θ_min_ = 1.9°
Absorption correction: multi-scan (*SADABS*; Bruker, 2009)	*h* = −21→21
*T*_min_ = 0.832, *T*_max_ = 0.924	*k* = −24→23
29877 measured reflections	*l* = −14→14

### Refinement

**Table:**

Refinement on *F*^2^	Primary atom site location: structure-invariant direct methods
Least-squares matrix: full	Secondary atom site location: difference Fourier map
*R*[*F*^2^ > 2σ(*F*^2^)] = 0.049	Hydrogen site location: inferred from neighbouring sites
*wR*(*F*^2^) = 0.159	H-atom parameters constrained
*S* = 1.05	*w* = 1/[σ^2^(*F*_o_^2^) + (0.075*P*)^2^ + 0.4271*P*] where *P* = (*F*_o_^2^ + 2*F*_c_^2^)/3
7900 reflections	(Δ/σ)_max_ < 0.001
290 parameters	Δρ_max_ = 0.53 e Å^−^^3^
0 restraints	Δρ_min_ = −0.45 e Å^−^^3^

### Special details

**Table:**

Geometry. All e.s.d.'s (except the e.s.d. in the dihedral angle between two l.s. planes) are estimated using the full covariance matrix. The cell e.s.d.'s are taken into account individually in the estimation of e.s.d.'s in distances, angles and torsion angles; correlations between e.s.d.'s in cell parameters are only used when they are defined by crystal symmetry. An approximate (isotropic) treatment of cell e.s.d.'s is used for estimating e.s.d.'s involving l.s. planes.
Refinement. Refinement of *F*^2^ against ALL reflections. The weighted *R*-factor *wR* and goodness of fit *S* are based on *F*^2^, conventional *R*-factors *R* are based on *F*, with *F* set to zero for negative *F*^2^. The threshold expression of *F*^2^ > σ(*F*^2^) is used only for calculating *R*-factors(gt) *etc*. and is not relevant to the choice of reflections for refinement. *R*-factors based on *F*^2^ are statistically about twice as large as those based on *F*, and *R*- factors based on ALL data will be even larger.

### Fractional atomic coordinates and isotropic or equivalent isotropic displacement parameters (Å^2^)

**Table:**

	*x*	*y*	*z*	*U*_iso_*/*U*_eq_	
Cl1	0.90496 (5)	0.48639 (5)	1.17911 (9)	0.1088 (3)	
S1	0.39474 (3)	0.45413 (3)	0.74551 (4)	0.05346 (13)	
S2	0.00345 (5)	0.24018 (6)	0.06766 (9)	0.1109 (3)	
O1	0.58819 (9)	0.47636 (7)	0.90982 (12)	0.0551 (3)	
N1	0.59354 (9)	0.34760 (8)	0.99633 (14)	0.0483 (3)	
H1N1	0.5589	0.2988	0.9997	0.058*	
N2	0.48488 (9)	0.33585 (8)	0.61649 (13)	0.0469 (3)	
N3	0.48455 (9)	0.30835 (8)	0.48666 (14)	0.0467 (3)	
N4	0.38196 (8)	0.40807 (7)	0.48700 (12)	0.0393 (3)	
C1	0.74454 (12)	0.40977 (11)	1.08375 (18)	0.0534 (4)	
H1A	0.7464	0.4416	1.0081	0.064*	
C2	0.81439 (13)	0.41592 (12)	1.1889 (2)	0.0623 (5)	
C3	0.81517 (16)	0.36979 (15)	1.3012 (2)	0.0758 (6)	
H3A	0.8632	0.3754	1.3704	0.091*	
C4	0.74302 (16)	0.31485 (16)	1.3089 (2)	0.0766 (6)	
H4A	0.7421	0.2828	1.3844	0.092*	
C5	0.67174 (13)	0.30652 (12)	1.20593 (19)	0.0598 (4)	
H5A	0.6239	0.2684	1.2119	0.072*	
C6	0.67148 (11)	0.35481 (10)	1.09415 (16)	0.0449 (3)	
C7	0.55154 (11)	0.41050 (10)	0.92502 (14)	0.0420 (3)	
C8	0.44971 (11)	0.39121 (11)	0.87723 (15)	0.0469 (3)	
H8A	0.4132	0.3957	0.9520	0.056*	
H8B	0.4462	0.3346	0.8479	0.056*	
C9	0.42302 (10)	0.39516 (9)	0.61317 (15)	0.0410 (3)	
C10	0.42372 (10)	0.35210 (9)	0.41135 (15)	0.0411 (3)	
C11	0.40297 (11)	0.34559 (11)	0.26504 (15)	0.0457 (3)	
H11A	0.4454	0.3056	0.2332	0.055*	
H11B	0.4161	0.3980	0.2260	0.055*	
C12	0.30248 (11)	0.32128 (10)	0.21711 (15)	0.0429 (3)	
C13	0.26102 (13)	0.25277 (12)	0.2653 (2)	0.0605 (5)	
H13A	0.2949	0.2215	0.3308	0.073*	
C14	0.17027 (15)	0.22984 (13)	0.2183 (2)	0.0679 (5)	
H14A	0.1439	0.1834	0.2525	0.081*	
C15	0.11798 (13)	0.27517 (13)	0.1210 (2)	0.0615 (5)	
C16	0.15828 (14)	0.34441 (14)	0.0742 (2)	0.0656 (5)	
H16A	0.1239	0.3765	0.0104	0.079*	
C17	0.24971 (13)	0.36663 (12)	0.12152 (17)	0.0549 (4)	
H17A	0.2760	0.4132	0.0879	0.066*	
C18	0.30298 (10)	0.46012 (9)	0.44437 (14)	0.0411 (3)	
C19	0.21336 (12)	0.43210 (13)	0.4587 (2)	0.0587 (4)	
H19A	0.2049	0.3821	0.4996	0.070*	
C20	0.13671 (15)	0.47902 (18)	0.4118 (3)	0.0819 (7)	
H20A	0.0760	0.4605	0.4207	0.098*	
C21	0.14890 (19)	0.55153 (18)	0.3532 (3)	0.0894 (8)	
H21A	0.0965	0.5827	0.3219	0.107*	
C22	0.2370 (2)	0.57963 (15)	0.3394 (3)	0.0892 (8)	
H22A	0.2444	0.6300	0.2991	0.107*	
C23	0.31656 (15)	0.53345 (12)	0.3851 (2)	0.0634 (5)	
H23A	0.3770	0.5522	0.3753	0.076*	
C24	−0.04042 (19)	0.3111 (2)	−0.0555 (3)	0.1131 (11)	
H24A	−0.1055	0.2992	−0.0837	0.170*	
H24B	−0.0045	0.3070	−0.1298	0.170*	
H24C	−0.0351	0.3655	−0.0199	0.170*	

### Atomic displacement parameters (Å^2^)

**Table:**

	*U*^11^	*U*^22^	*U*^33^	*U*^12^	*U*^13^	*U*^23^
Cl1	0.0754 (4)	0.0927 (5)	0.1462 (7)	−0.0411 (3)	−0.0421 (4)	0.0276 (4)
S1	0.0579 (2)	0.0499 (2)	0.0494 (2)	0.01375 (18)	−0.00817 (17)	−0.00946 (17)
S2	0.0727 (4)	0.1321 (7)	0.1206 (6)	−0.0488 (4)	−0.0226 (4)	0.0262 (5)
O1	0.0541 (7)	0.0477 (6)	0.0610 (7)	−0.0125 (5)	−0.0046 (5)	0.0102 (5)
N1	0.0456 (7)	0.0414 (7)	0.0549 (8)	−0.0092 (5)	−0.0071 (6)	0.0032 (6)
N2	0.0430 (6)	0.0449 (7)	0.0501 (7)	0.0090 (5)	−0.0070 (5)	−0.0012 (5)
N3	0.0435 (6)	0.0438 (7)	0.0514 (7)	0.0092 (5)	−0.0011 (5)	−0.0027 (5)
N4	0.0355 (5)	0.0386 (6)	0.0422 (6)	0.0057 (5)	−0.0031 (4)	0.0006 (5)
C1	0.0454 (8)	0.0511 (10)	0.0610 (10)	−0.0068 (7)	−0.0060 (7)	0.0050 (8)
C2	0.0498 (9)	0.0520 (10)	0.0803 (13)	−0.0091 (8)	−0.0139 (8)	0.0001 (9)
C3	0.0683 (12)	0.0763 (14)	0.0750 (14)	−0.0042 (11)	−0.0275 (10)	0.0053 (11)
C4	0.0702 (13)	0.0887 (16)	0.0660 (12)	−0.0067 (12)	−0.0148 (10)	0.0232 (11)
C5	0.0512 (9)	0.0600 (11)	0.0660 (11)	−0.0051 (8)	−0.0036 (8)	0.0141 (9)
C6	0.0418 (7)	0.0409 (8)	0.0506 (8)	−0.0007 (6)	−0.0018 (6)	−0.0011 (6)
C7	0.0441 (7)	0.0438 (8)	0.0374 (7)	−0.0051 (6)	0.0011 (5)	−0.0029 (6)
C8	0.0456 (8)	0.0518 (9)	0.0417 (7)	−0.0062 (7)	−0.0027 (6)	−0.0006 (6)
C9	0.0374 (6)	0.0390 (7)	0.0444 (7)	0.0031 (5)	−0.0055 (5)	−0.0004 (6)
C10	0.0369 (6)	0.0397 (7)	0.0460 (7)	0.0030 (5)	0.0018 (5)	0.0000 (6)
C11	0.0423 (7)	0.0505 (9)	0.0445 (8)	0.0019 (6)	0.0053 (6)	−0.0005 (6)
C12	0.0438 (7)	0.0431 (8)	0.0416 (7)	0.0019 (6)	0.0048 (6)	−0.0021 (6)
C13	0.0519 (9)	0.0537 (10)	0.0751 (12)	0.0041 (8)	0.0031 (8)	0.0194 (9)
C14	0.0589 (10)	0.0519 (11)	0.0925 (15)	−0.0084 (9)	0.0071 (10)	0.0148 (10)
C15	0.0524 (9)	0.0702 (12)	0.0605 (10)	−0.0134 (9)	−0.0004 (8)	−0.0018 (9)
C16	0.0596 (10)	0.0783 (13)	0.0546 (10)	−0.0111 (9)	−0.0126 (8)	0.0179 (9)
C17	0.0555 (9)	0.0586 (10)	0.0485 (9)	−0.0116 (8)	−0.0032 (7)	0.0109 (7)
C18	0.0399 (7)	0.0406 (7)	0.0410 (7)	0.0090 (6)	−0.0034 (5)	0.0006 (6)
C19	0.0411 (8)	0.0640 (11)	0.0698 (11)	0.0058 (8)	0.0014 (7)	0.0052 (9)
C20	0.0452 (10)	0.1068 (19)	0.0905 (16)	0.0244 (11)	−0.0069 (10)	−0.0045 (14)
C21	0.0787 (16)	0.0984 (19)	0.0846 (16)	0.0471 (14)	−0.0195 (12)	−0.0029 (14)
C22	0.120 (2)	0.0542 (12)	0.0880 (16)	0.0259 (13)	−0.0135 (15)	0.0193 (11)
C23	0.0667 (11)	0.0490 (10)	0.0731 (12)	0.0034 (8)	0.0010 (9)	0.0127 (8)
C24	0.0639 (14)	0.157 (3)	0.111 (2)	−0.0165 (17)	−0.0253 (14)	0.011 (2)

### Geometric parameters (Å, °)

**Table:**

Cl1—C2	1.740 (2)	C10—C11	1.487 (2)
S1—C9	1.7371 (16)	C11—C12	1.514 (2)
S1—C8	1.7964 (16)	C11—H11A	0.9700
S2—C15	1.7574 (19)	C11—H11B	0.9700
S2—C24	1.767 (3)	C12—C17	1.377 (2)
O1—C7	1.2128 (19)	C12—C13	1.380 (2)
N1—C7	1.357 (2)	C13—C14	1.380 (3)
N1—C6	1.411 (2)	C13—H13A	0.9300
N1—H1N1	0.9408	C14—C15	1.385 (3)
N2—C9	1.3075 (19)	C14—H14A	0.9300
N2—N3	1.3927 (19)	C15—C16	1.376 (3)
N3—C10	1.3038 (19)	C16—C17	1.386 (3)
N4—C9	1.3650 (18)	C16—H16A	0.9300
N4—C10	1.3717 (19)	C17—H17A	0.9300
N4—C18	1.4383 (17)	C18—C23	1.364 (2)
C1—C2	1.380 (2)	C18—C19	1.380 (2)
C1—C6	1.388 (2)	C19—C20	1.375 (3)
C1—H1A	0.9300	C19—H19A	0.9300
C2—C3	1.366 (3)	C20—C21	1.345 (4)
C3—C4	1.374 (3)	C20—H20A	0.9300
C3—H3A	0.9300	C21—C22	1.358 (4)
C4—C5	1.381 (3)	C21—H21A	0.9300
C4—H4A	0.9300	C22—C23	1.398 (3)
C5—C6	1.382 (2)	C22—H22A	0.9300
C5—H5A	0.9300	C23—H23A	0.9300
C7—C8	1.512 (2)	C24—H24A	0.9600
C8—H8A	0.9700	C24—H24B	0.9600
C8—H8B	0.9700	C24—H24C	0.9600
			
C9—S1—C8	98.04 (7)	C10—C11—H11B	108.6
C15—S2—C24	104.42 (12)	C12—C11—H11B	108.6
C7—N1—C6	125.38 (13)	H11A—C11—H11B	107.6
C7—N1—H1N1	117.3	C17—C12—C13	117.67 (15)
C6—N1—H1N1	114.9	C17—C12—C11	120.62 (15)
C9—N2—N3	106.43 (12)	C13—C12—C11	121.71 (15)
C10—N3—N2	108.13 (12)	C14—C13—C12	121.26 (17)
C9—N4—C10	104.81 (11)	C14—C13—H13A	119.4
C9—N4—C18	127.86 (12)	C12—C13—H13A	119.4
C10—N4—C18	126.78 (12)	C13—C14—C15	120.78 (18)
C2—C1—C6	118.11 (17)	C13—C14—H14A	119.6
C2—C1—H1A	120.9	C15—C14—H14A	119.6
C6—C1—H1A	120.9	C16—C15—C14	118.25 (17)
C3—C2—C1	122.97 (18)	C16—C15—S2	124.72 (16)
C3—C2—Cl1	118.34 (15)	C14—C15—S2	117.02 (15)
C1—C2—Cl1	118.68 (16)	C15—C16—C17	120.50 (18)
C2—C3—C4	118.09 (18)	C15—C16—H16A	119.8
C2—C3—H3A	121.0	C17—C16—H16A	119.8
C4—C3—H3A	121.0	C12—C17—C16	121.53 (17)
C3—C4—C5	120.9 (2)	C12—C17—H17A	119.2
C3—C4—H4A	119.5	C16—C17—H17A	119.2
C5—C4—H4A	119.5	C23—C18—C19	121.08 (16)
C4—C5—C6	120.03 (18)	C23—C18—N4	120.47 (15)
C4—C5—H5A	120.0	C19—C18—N4	118.37 (14)
C6—C5—H5A	120.0	C20—C19—C18	119.2 (2)
C5—C6—C1	119.86 (15)	C20—C19—H19A	120.4
C5—C6—N1	117.46 (15)	C18—C19—H19A	120.4
C1—C6—N1	122.64 (15)	C21—C20—C19	120.4 (2)
O1—C7—N1	124.62 (14)	C21—C20—H20A	119.8
O1—C7—C8	123.76 (15)	C19—C20—H20A	119.8
N1—C7—C8	111.39 (13)	C20—C21—C22	120.6 (2)
C7—C8—S1	116.35 (12)	C20—C21—H21A	119.7
C7—C8—H8A	108.2	C22—C21—H21A	119.7
S1—C8—H8A	108.2	C21—C22—C23	120.6 (2)
C7—C8—H8B	108.2	C21—C22—H22A	119.7
S1—C8—H8B	108.2	C23—C22—H22A	119.7
H8A—C8—H8B	107.4	C18—C23—C22	118.1 (2)
N2—C9—N4	110.87 (13)	C18—C23—H23A	121.0
N2—C9—S1	127.18 (11)	C22—C23—H23A	121.0
N4—C9—S1	121.93 (11)	S2—C24—H24A	109.5
N3—C10—N4	109.75 (13)	S2—C24—H24B	109.5
N3—C10—C11	126.37 (14)	H24A—C24—H24B	109.5
N4—C10—C11	123.86 (13)	S2—C24—H24C	109.5
C10—C11—C12	114.61 (13)	H24A—C24—H24C	109.5
C10—C11—H11A	108.6	H24B—C24—H24C	109.5
C12—C11—H11A	108.6		
			
C9—N2—N3—C10	0.44 (17)	C9—N4—C10—C11	−177.83 (14)
C6—C1—C2—C3	0.8 (3)	C18—N4—C10—C11	10.2 (2)
C6—C1—C2—Cl1	−178.42 (14)	N3—C10—C11—C12	117.85 (17)
C1—C2—C3—C4	0.1 (4)	N4—C10—C11—C12	−64.0 (2)
Cl1—C2—C3—C4	179.4 (2)	C10—C11—C12—C17	128.51 (17)
C2—C3—C4—C5	0.0 (4)	C10—C11—C12—C13	−52.5 (2)
C3—C4—C5—C6	−1.1 (4)	C17—C12—C13—C14	0.8 (3)
C4—C5—C6—C1	2.1 (3)	C11—C12—C13—C14	−178.17 (18)
C4—C5—C6—N1	−175.7 (2)	C12—C13—C14—C15	0.0 (3)
C2—C1—C6—C5	−1.9 (3)	C13—C14—C15—C16	−1.2 (3)
C2—C1—C6—N1	175.77 (17)	C13—C14—C15—S2	179.65 (18)
C7—N1—C6—C5	141.23 (18)	C24—S2—C15—C16	1.5 (3)
C7—N1—C6—C1	−36.5 (3)	C24—S2—C15—C14	−179.4 (2)
C6—N1—C7—O1	20.1 (3)	C14—C15—C16—C17	1.6 (3)
C6—N1—C7—C8	−154.53 (15)	S2—C15—C16—C17	−179.32 (17)
O1—C7—C8—S1	22.1 (2)	C13—C12—C17—C16	−0.4 (3)
N1—C7—C8—S1	−163.17 (12)	C11—C12—C17—C16	178.58 (18)
C9—S1—C8—C7	90.32 (13)	C15—C16—C17—C12	−0.8 (3)
N3—N2—C9—N4	−0.06 (17)	C9—N4—C18—C23	104.7 (2)
N3—N2—C9—S1	−178.58 (12)	C10—N4—C18—C23	−85.2 (2)
C10—N4—C9—N2	−0.32 (17)	C9—N4—C18—C19	−78.7 (2)
C18—N4—C9—N2	171.54 (14)	C10—N4—C18—C19	91.5 (2)
C10—N4—C9—S1	178.29 (11)	C23—C18—C19—C20	0.3 (3)
C18—N4—C9—S1	−9.9 (2)	N4—C18—C19—C20	−176.41 (18)
C8—S1—C9—N2	−14.31 (16)	C18—C19—C20—C21	−0.3 (4)
C8—S1—C9—N4	167.32 (13)	C19—C20—C21—C22	0.0 (4)
N2—N3—C10—N4	−0.65 (17)	C20—C21—C22—C23	0.4 (4)
N2—N3—C10—C11	177.73 (14)	C19—C18—C23—C22	0.1 (3)
C9—N4—C10—N3	0.60 (17)	N4—C18—C23—C22	176.72 (19)
C18—N4—C10—N3	−171.37 (14)	C21—C22—C23—C18	−0.4 (4)

### Hydrogen-bond geometry (Å, °)

**Table:**

*D*—H···*A*	*D*—H	H···*A*	*D*···*A*	*D*—H···*A*
N1—H1N1···N3^i^	0.94	2.04	2.9787 (19)	174
C8—H8A···O1^ii^	0.97	2.52	3.147 (2)	123
C11—H11B···O1^iii^	0.97	2.47	3.416 (2)	165

Symmetry codes: (i) *x*, −*y*+1/2, *z*+1/2; (ii) −*x*+1, −*y*+1, −*z*+2; (iii) −*x*+1, −*y*+1, −*z*+1.
